# Development and Application of Droplet Digital PCR Tools for the Detection of Transgenes in Pastures and Pasture-Based Products

**DOI:** 10.3389/fpls.2018.01923

**Published:** 2019-01-08

**Authors:** Paula A. Giraldo, Noel O. I. Cogan, German C. Spangenberg, Kevin F. Smith, Hiroshi Shinozuka

**Affiliations:** ^1^Faculty of Veterinary and Agricultural Sciences, The University of Melbourne, Parkville, VIC, Australia; ^2^Agriculture Victoria, AgriBio, Centre for AgriBioscience, Bundoora, VIC, Australia; ^3^School of Applied Systems Biology, La Trobe University, Bundoora, VIC, Australia; ^4^Agriculture Victoria, Hamilton, VIC, Australia

**Keywords:** genetically modified (GM), forage, real-time PCR (qPRC), droplet digital PCR (ddPCR), TaqMan-probe, SYBR Green I

## Abstract

Implementation of molecular biotechnology, such as transgenic technologies, in forage species can improve agricultural profitability through achievement of higher productivity, better use of resources such as soil nutrients, water, or light, and reduced environmental impact. Development of detection and quantification techniques for genetically modified plants are necessary to comply with traceability and labeling requirements prior to regulatory approval for release. Real-time PCR has been the standard method used for detection and quantification of genetically modified events, and droplet digital PCR is a recent alternative technology that offers a higher accuracy. Evaluation of both technologies was performed using a transgenic high-energy forage grass as a case study. Two methods for detection and quantification of the transgenic cassette, containing modified fructan biosynthesis genes, and a selectable marker gene, hygromycin B phosphotransferase used for transformation, were developed. Real-time PCR was assessed using two detection techniques, SYBR Green I and fluorescent probe-based methods. A range of different agricultural commodities were tested including fresh leaves, tillers, seeds, pollen, silage and hay, simulating a broad range of processed agricultural commodities that are relevant in the commercial use of genetically modified pastures. The real-time and droplet digital PCR methods were able to detect both exogenous constructs in all agricultural products. However, a higher sensitivity and repeatability in transgene detection was observed with the droplet digital PCR technology. Taking these results more broadly, it can be concluded that the droplet digital PCR technology provides the necessary resolution for quantitative analysis and detection, allowing absolute quantification of the target sequence at the required limits of detection across all jurisdictions globally. The information presented here provides guidance and resources for pasture-based biotechnology applications that are required to comply with traceability requirements.

## Introduction

Grasslands are among the largest ecosystems on earth, compromising 35% of the global land area, compared with 12% used for cultivation of agricultural crops (Dubois, 2011). Implementation of transgenic technologies in forage species can improve agriculture through higher productivity, better use of resources and reduced environmental impact. Genetic solutions for forage quality limitations, pest and disease resistance, nutrient acquisition efficiency, tolerance to abiotic stresses and the targeted modification of growth and development, can be achieved by introducing novel high impact traits into forage breeding programs (Smith et al., 2007).

All new genetically modified (GM) cultivars are required to be assessed for regulatory requirement purposes prior to commercial release, which aims to provide an evaluation of their potential impacts on human, animal and environmental health. Establishment of tracking and tracing tools for the transgene insertion is an essential part of the deregulation process. Detection methods for GM identification and quantification, are not only important to ensure legality and traceability, but also to comply with GM labeling regulations (European Parliament, 2003).

To date assessment of GM crops has focused on the plant product that is going to be used for human consumption. For instance, grains of GM maize, beans of GM soybean, and seeds of GM rapeseed and cotton. However, between 70 and 90% of all GM crops and their biomass are used in farm as animal feed (Flachowsky et al., 2012). The European Food Safety Authority (EFSA) recently have acknowledged the need for clarification on the safety assessment of GM feed of plant origin and published an explanatory note providing a forage definition for the major GM commercial crops (maize, soybean, sugarbeet, rapeseed, and cotton) (European Food Safety Authority [EFSA] et al., 2018).

Currently, most GM event detection and quantification methods used by national reference laboratories are developed and optimized for a real-time PCR (qPCR) platform. Nevertheless, qPCR has some notable drawbacks, such as the negative impact of inhibitors in the amplification efficiency, which represent challenges for applicants during the GM de-regulations process. Additionally, the requirement of reference material to use in calibrations, which is rarely commercially available, especially for niche transgenic cassettes or sequences and unauthorized events is another downside.

Emerging PCR-based technologies, such that droplet digital PCR (ddPCR), can overcome those obstacles. This technology relies on the same DNA amplification principles as the standard PCR and qPCR, but works through partitioning PCR mix into 20,000 nanoliter-sized droplets. Features such as absolute quantification, avoidance of using standard curves, high resilience to inhibitors leading to a less restrictive amplification efficiency, make ddPCR a promising alternative for GM event detection (Rački et al., 2014; Corbisier et al., 2015).

In temperate forage species that are being studied for potential GM-based improvement, qPCR could be the preferred technique to deliver the necessary GM traceability. The most relevant forage species in temperate areas have been the subject of active research into the development of transgenic cultivars including grasses such as perennial ryegrass (*Lolium perenne* L.) (Badenhorst et al., 2018), and tall fescue (*Festuca arundinacea* Schreb.) and legumes such as white clover (*Trifolium repens L.*) (Panter et al., 2012) and red clover (*Trifolium pratense* L.) (Wang and Brummer, 2012). The “Roundup Ready” cultivar of alfalfa (*Medicago sativa* L.) is the first commercially available transgenic forage, and issues relating to the detection of the transgene and market co-existence have been addressed (Putnam et al., 2016).

A transgenic high-energy perennial ryegrass was selected as case study for the evaluation and comparison of qPCR and ddPCR. A targeted up-regulation of fructan biosynthesis in the leaf blades of perennial ryegrass was obtained by re-programming the expression of fructan biosynthesis genes through the transgenic manipulation of 6-glucose fructosyltransferase (6G-FFT) and sucrose:sucrose 1-fructosyl-transferase (1SST) (Panter et al., 2017). During the transformation of high-energy ryegrass an antibiotic resistance factor, hygromycin B phosphotransferase (*hph*) gene, was introduced as a selectable marker.

For detection of GM sequences from plants, a sensitive and reliable endogenous reference gene is required as an experimental control. Such reference gene should be ideally single-copy within the genome, and non-variant in copy number across the cultivars and species (Xue et al., 2014). Due to several whole genome duplications in the evolutionary history of flowering plants (Angiosperm), crop genomes typically exhibit complex structure and genetic redundancy making the identification of the optimal reference gene more complex (Ren et al., 2018). Plant Cullin4 (Cul4) genes are potential candidates, because they are relatively highly conserved between species, and an *in silico* analysis suggested the single copy status of the gene in a range of flowering plants (Marín, 2009).

Additionally, GM detection must not only identify the presence of the transgenic sequence in a low concentration, but also be accurately identified in all different agricultural commodities the species in question generates across the agricultural supply chain (Cankar et al., 2006). For instance, in forage legumes these include fresh leaves, dry leaves, pollen, seeds, stems, hay and honey (Panter et al., 2015). In wind pollinated grass species used for grazing fresh leaves, dry leaves, pollen, seeds, stems, hay and silage are target products based on their role in agricultural production systems or relevance in a co-existence framework (Smith and Spangenberg, 2016). Adequate sampling protocols must be developed in conjunction with appropriate and validated methods of extraction, amplification and detection of the possible exogenous GM sequences.

In the present study, evaluation of the common qPCR and new ddPCR-based transgene detection techniques in relevant agricultural commodities of GM forage crops is discussed. Transgenic high-energy ryegrass was selected because it exemplifies one of the most complex scenarios for GM crops tracking and tracing purposes; the transgene is composed of endogenous genes (cisgene) and the sequence composition is highly skewed toward to guanine and cytosine (GC rich). Additionally, *Lp*Cul4, a single copy endogenous gene of perennial ryegrass, is reported for first time.

## Materials and Methods

### Experimental Materials

All plant materials were maintained at the Agriculture Victoria Research Hamilton centre (Department of Economic Development, Jobs Transport and Resources). For the transformation, perennial ryegrass variety FLP418-20, was selected for use as donor material, based on the observed shoot regeneration from embryogenic callus (EC) derived from mature seeds of FLP418 (PGG Wrightson Seeds, Christchurch, New Zealand). Clonal replicates of the genotype FLp418-20, were subjected to transformation using biolistic-mediated DNA delivery. A detailed description of the transgenic ryegrass plants generation is published in Panter et al. (2017). The transgenic event 10 used in this experiment was hemizygous for the transgene, and the non-transgenic material was its null segregants (a genotype from perennial ryegrass plant FLP418-20). All transgenic and non-transgenic plants were grown under physical containment level 2 glasshouse conditions.

#### Raw Material

Fresh leaves, tillers, seeds and pollen (ca. 0.5 g) of transgenic perennial ryegrass and its null segregants (negative control), were harvested into 50 mL falcon tubes. Pollen grains were isolated from ryegrass inflorescences using a method adapted from Becker et al. (2003). Briefly, mature ryegrass inflorescences in which the terminal florets had not yet opened were collected into a 50 mL plastic falcon tube, and 2 mL of distilled water per inflorescence was added to the tube, which was then agitated to release the pollen from the anthers. Aliquots of 1 mL of each sample were transferred to 1.5 mL microcentrifuge tubes and pollen grains were precipitated by centrifugation.

#### Conserved Material

To produce hay or air-dried mature herbage, approximately 10 g of fresh material between head emerge but prior to flowering was harvested into paper bags, distributed evenly through the bags and dried in a horizontal position for 72 h on raised wire racks in a growth chamber, with 16 h at 25°C (day) and 8 h at 18°C (night). To produce silage, approximately 100 g of fresh plant material were harvested, ensiled in vacuum bags, and storage in a dark place at room temperature (22°C ± 2°C). After 4 weeks of fermentation, vacuum bags were unsealed and stored at -80°C.

### Experimental Methods

#### DNA Extraction

DNA was extracted from 20 biological replicates of the untransformed perennial ryegrass genotype (FLP 418-20), and event 10 plants. Six different agricultural products were analyzed for a total of 240 DNA extractions. All sample materials were freeze-dried for 48 h with the freeze-dry system, FreeZone 4.5 Liter Benchtop Freeze Dry System instrument (Labconco, Kansas City, MO, United States) and ground to a fine powder using the TissueLyser II instrument (Qiagen, Hilden, Germany). Genomic DNA was extracted using the DNeasy^TM^ Plant Mini Kit (Qiagen), following manufactures’ instructions. DNA concentrations were measured using the NanoDrop 1000 UV-vis spectrophotometer (Thermo Fisher Scientific, Waltham, MA, United States) and DNA concentrations were normalized to 10 ng/μl.

#### Assay Design

**FIGURE 1:**
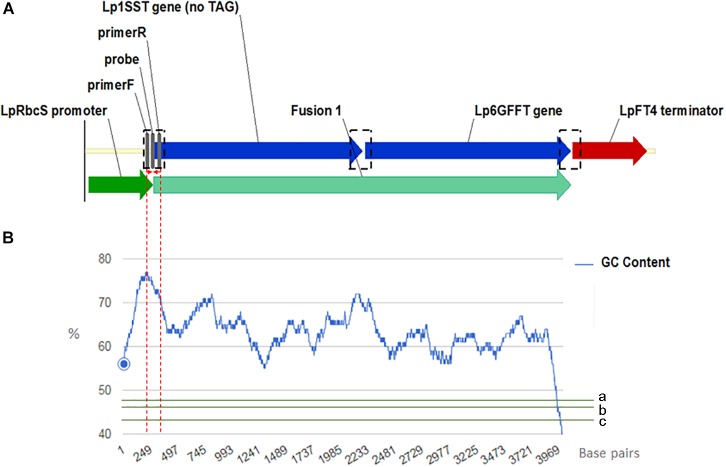
Frame of transgenic elements in the event 10 ryegrass genome **(A)**. Location of the designed construct-specific primer pairs and probe targeting the transgene are in gray color. The transcriptional direction is indicated with arrow, and the perennial ryegrass rubisco promoter, 1SST-6G-FFT fusion protein gene, and FT4 terminator sequences are shown with dark green, blue, and red arrows, respectively. in black dotted rectangles possible location for the construct specific primers and probe. For designing the SST-FFT fusion protein gene, the terminal codon (TAG) of the perennial ryegrass SST gene was removed. GC content distribution **(B)** of 1SST-6G-FFT fusion protein gene in blue, compare with rice genic (a), rice genome average (b) and rice intergenic (c) in green. Red dotted lines showed a 200 base-pairs window where the set of primers and probe are located.

Manual sequence analysis and primer design was performed using Sequencher version 5.0.1 (GeneCodes, Ann Arbor, MI, United States) and Primer3.^1^ PCR primers and probes for the perennial ryegrass Cullin 4 gene, *Lp*Cul4 (Marín, 2009), the transgenic cassette containing modified fructan biosynthesis genes (1SST-6G-FFT; Panter et al., 2017), and the *hph* gene (Blochlinger and Diggelmann, 1984) were designed in this study and tested to generate amplicons shorter than 201 base pairs in length (Supplementary Table S3). The same primer and probe sets were used for both qPCR (SYBR Green I-based and probe-based) and ddPCR assays. The vector, possible and actual location of the designed construct-specific primer pairs and probe targeting the transgene are shown in Figure 1A, as well as GC content distribution of the transgenic cassette (Figure 1B). All primers and probes were synthesized at Integrated DNA Technologies Pte. (Singapore Science Park II, Singapore). Before detection and quantification of endo and exogenous genes, amplification efficiency and reproducibility for each primer set were examined through a standard curve assay, using DNA dilutions of plasmid DNA in the case of the exogenous genes, and genomic DNA for the reference gene. For ddPCR, assays were optimized using a thermal protocol with a range of annealing temperatures (55–65°C). Each PCR-based assay was performed in four technical replicates.

#### Real-Time PCR

Reactions were prepared using the SsoAdvanced^TM^ Universal Supermix kit (Bio-Rad Laboratories, Hercules, CA, United States) for probes and SYBR^®^ Green I. PCR reaction (total volume: 20 μl) consisted of 20 ng (in 4 μl) DNA template, 1 μl each of the forward and reverse primer adjusted to 10 μM, 10 μl SsoAdvanced^TM^ Universal Supermix (2x), and for the probe-base assay 0.5 μl target-gene probe adjusted to 10 μM. All reactions were performed using a CFX connect qPCR instrument (Bio-Rad Laboratories) using the following program; initial denaturation of template DNA at 95°C for 30 s, followed by 40 cycles of amplification reaction (20 s 95°C; 30 s 60°C). Genomic DNA fragments and DNA plasmids (described above) were used as positive control (PC) templates for amplification, along with no-template controls (NTC). For each tissue (6 in total), 20 biological replicates of the isogenic control (FLP 481-20) and 20 of event10 were analyzed within four technical replicates.

#### Digital-Droplet PCR

The ddPCR Supermix for Probes (no UTP) kit (Bio-Rad Laboratories) was used as the basis for all reactions, the primers and probes was adjusted to a concentration of 100 μM. Following manufacturer’s instruction, a total volume of 22 μl was prepared, containing; 0.12 μl target-gene forward primer, 0.12 μl target-gene reverse primer, 0.024 μl target-gene probe, 0.12 μl reference-gene forward primer, 0.12 μl reference-gene reverse primer, 0.024 μl reference-gene probe, 12 μl ddPCR Supermix for Probes (2x), and 9.472 μl distilled water. To each solution, 20 ng DNA template was added, nanoliter-sized droplets were generated on the AutoDG^TM^ Instrument (Bio-Rad Laboratories), following manufacturer’s instruction. PCR amplification was performed with a T100 PCR Thermal Cycler (Bio-Rad Laboratories), with the following temperature profile: 10 min at 95°C for initial denaturation, 40 cycles of 95°C for 30 s, and 60°C for 60 s, followed by 98°C for 10 min. After PCR cycling was complete, the reactions were placed in a QX200 instrument (Bio-Rad Laboratories) and droplets were analyzed according to manufacturer’s instructions. For each tissue (6 in total), 3 biological replicates of the isogenic control (FLP 481-20) and 3 of event10 were analyzed within 3 technical replicates.

#### Data Analysis

qPCR raw data were processed using BioRad CFX Manager 3.1. The cycle threshold (Ct) value denotes the cycle at which the fluorescent signal first showed significant difference with respect to the background. All biological and technical replicates were used to calculate the average Ct value. Relative copy number of the target gene (1SST-6G-FFT) was calculated using the comparative ΔΔCt method with *Lp*Cul4 as reference gene. ddPCR data was analyzed with the QuantaSoft software versions 1.3.2.0 (Bio-Rad Laboratories).

## Results

### DNA Extraction

At the end of the extraction procedure, purified DNA was eluted with 100 μl of the AE buffer, and a subsequent measurement indicated that the concentrations varied between 4.8 and 83.25 ng/μl, and absorbance ratio (260/280) were between 1.66 and 1.85. On average, DNA concentrations from fresh leaves, tiller, seeds, pollen, silage and hay were 83.2, 23.8, 30.47, 4.8, 48.3, and 79.27 ng/μl, respectively. The lowest yield was obtained from pollen samples (4.8 ng/μl), which required an additional purification and concentration step to reach an acceptable concentration. A relatively low DNA purity was observed from the silage sample on NanoDrop system (260/280 = 1.66).

### Assay Designing and Validation

**FIGURE 2:**
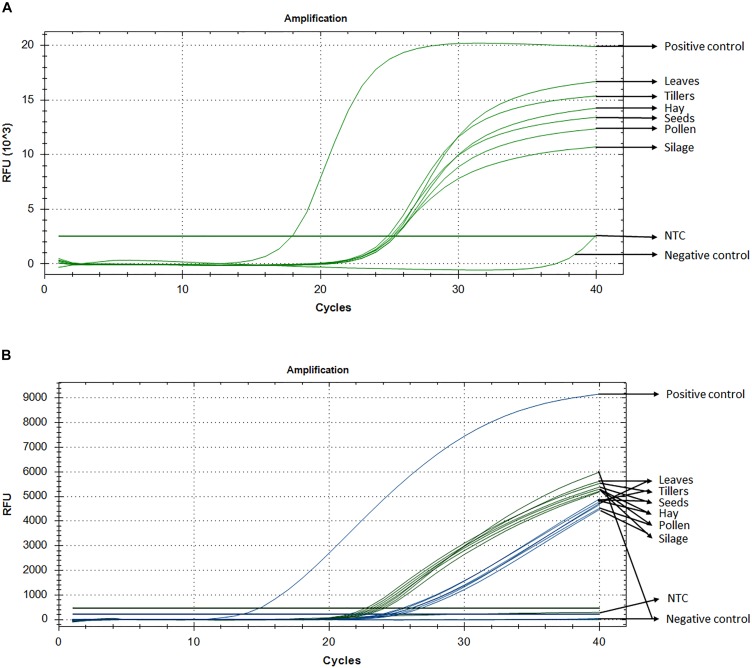
Detection and quantification of 1SST-6G-FFT construct, using qPCR with SYBR Green I **(A)** and fluorescent probe **(B)**. In probe-base assay the target construct (1SST-6G-FFT, FAM) is shown in blue and the reference gene (*Lp*Cul4, HEX) in green. *Y*-axis shows relative fluorescence unit (RFU), and the *X*-axis denotes PCR cycle number.

Standard curve assays were performed for each primer pair and probe sets, in both probe and SYBR Green I based assay. Amplification efficiencies using SYBR Green I fluorescence were between 95 and 107% (Supplementary Figure S1) and those of probe-based assay were between 104 and 81% for *Lp*Cul4 and 1SST-6G-FFT and 91 and 88% for *Lp*Cul4 and *hph*, respectively (Supplementary Figure S2). Additionally, melting curve assays were performed when using SYBR Green I fluorescence (Supplementary Figure S8). A gradient PCR was performed to identify that the optimal range of annealing temperatures was between 59°C and 61.2°C for all *Lp*Cul4, 1SST-6G-FFT and *hph* primers and probes (Supplementary Figure S3). Therefore, an optimized annealing temperature of 60°C was chosen for the subsequent experiments. Due to the similarity of the complete data set derived from the qPCR assays (both SYBR Green I and fluorescent probe-based), a single representative sample is presented (Figure 2A). And, Ct means, standard deviation and coefficient of variation for all tissues are presented in Supplementary Table S1.

### SYBR Green I-Based qPCR

Detection of the transgenic insertion 1SST-6G-FFT with SYBR Green I fluorescence was successfully achieved from all transgenic plant-derived DNA samples. The target sequence was amplified from PC (plasmid DNA) after 17 cycles, while amplification from transgenic plant-derived DNA samples started at around 25 cycles. Amplification from non-transgenic plant-derived DNA samples was observed only after 39 cycles. All Ct values (the cycle numbers in which fluorescent signals reached the threshold) from transgenic tissue were between 24.8 and 25.4 with the lowest value observed from hay and the highest from silage. Transgenic plant-derived DNA samples were differentiated from those of non-transgenic plants, with around 15 cycles (Figure 2). Detection of the selectable marker, *hph*, was also performed, and similar results were obtained. PC amplified earlier (Ct = 19.7), while transgenic tissues fluctuated between 24 and 25 cycle and non-transgenic tissue (negative control) amplified after 38 cycles (Supplementary Figure S4A). No significant amplification was observed from NTC, within 40 cycles.

### Probe-Based qPCR Assay

Probe and primer set for 1SST-6G-FFT insertion cassette (FAM fluorescence) and *Lp*Cul4 reference gene (HEX fluorescence), was tested with the PC (plasmid DNA containing the 1SST-6G-FFT sequence), transgenic tissue samples, and negative control (FLP418-20 genomic DNA). As expected, in the PC amplification was detected for 1SST-6G-FFT (Ct = 13.02) but not for *Lp*Cul4, whilst the negative control showed the opposite, amplification for *Lp*Cul4 (Ct = 22.16) and none for 1SST-6G-FFT (Figure 2B). Amplification from transgenic event 10 samples was detected with both fluorescence channels, presenting Ct values between 23.1 to 28.2 for *Lp*Cul4 (HEX) and 24.2 to 30.7 for 1SST-6G-FFT (FAM) (Figure 2B and Supplementary Table S1). There was a difference in 1SST-6G-FFT (FAM) Ct values between transgenic and non-transgenic plant-derived samples This trend was also observed with highly processed samples, such us silage, in which 4 cycle difference in Ct values were observed between transgenic and non-transgenic plants. qPCR probe-based assay could effectively detect event 10 transgenic material in all agricultural commodities evaluated. The same probe assay with six tissue samples was evaluated with *hph* and *Lp*Cul4 genes, obtaining similar results (Supplementary Figure S4B).

### ddPCR Assay

**FIGURE 3:**
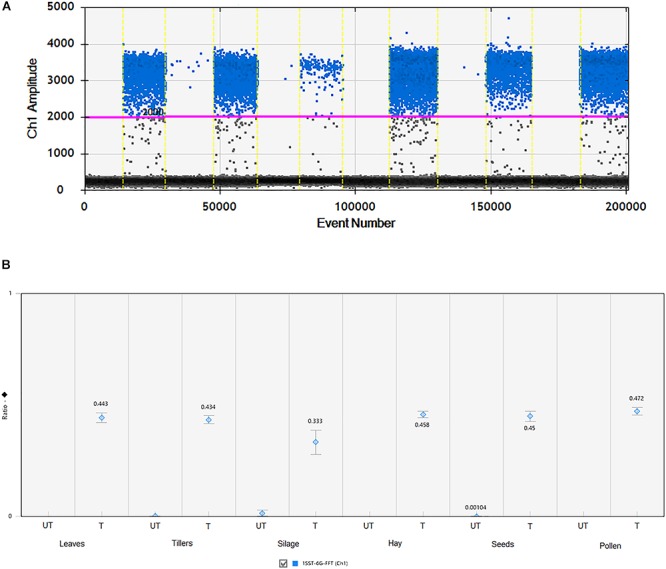
Detection and quantification of 1SST-6G-FFT construct (FAM in blue) using ddPCR. **(A)** 1D fluorescence amplitude plot, where set threshold is shown with a pink line, blue dots indicate presence of the 1SST-6G-FFT sequence in the droplet, and gray dots indicate absence of the sequence. **(B)** Ratio of 1SST-6G-FFT construct and *Lp*Cul4. UT and T stand for untransformed and transformed, respectively. Error bars indicate the Poisson 95% confidence intervals for each measurement.

All agricultural commodities were analyzed using the ddPCR technologies and results are presented in Figure 3 and Supplementary Table S2. Results showed that the 1SST-6G-FFT transgenic insertion is detected in the relevant samples, for instance when the sample was non-transgenic it presented only between 7 and 11 droplets, while transgenic samples showed between 161 and 1223 positive droplets (Figure 3 and Supplementary Table S2). Although, all DNA concentrations were normalized to 10 ng/μl, differences in the droplet counts for the transgenic insertion and reference gene were observable among tissues. From the DNA purity and quality evaluation the silage samples were identified as more degraded relative to the other tissues, but still there was a difference in count numbers between transgenic and non-transgenic silage. The ratio between the target genes (1SST-6G-FFT) and the endogenous gene (*Lp*Cul4) was relatively close to 0.5 for most of the tissue samples (0.434–0.472), except for silage (0.333), this was expected since the event 10 samples were hemizygous, and the *Lp*Cul4 probe was designed to detect both alleles of *Lp*Cul4 (two copies in the diploid perennial ryegrass genome). The same ddPCR assay was performed for the *hph* gene as the target gene and results are comparable with the 1SST-6G-FFT gene (Supplementary Figure S5).

### Copy Number Assay

**FIGURE 4:**
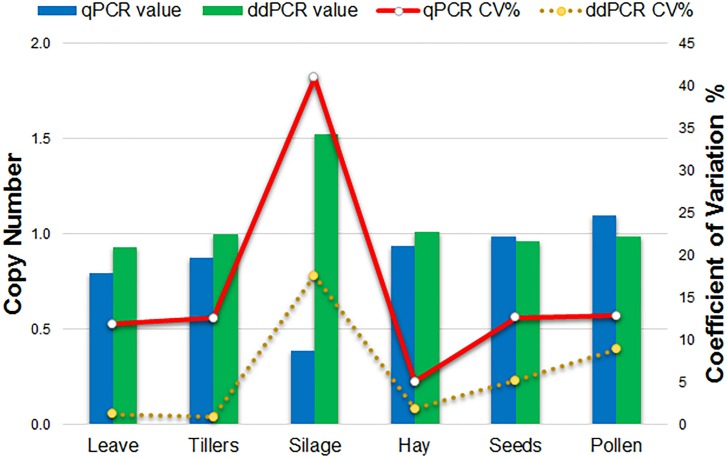
Comparison of qPCR and ddPCR in all agricultural commodities. Histograms indicate the average relative copy number in each tissue **(left scale)**. Lines show the trend of the variation (CV%) of the qPCR and ddPCR assays **(right scale).**

Comparison of qPCR and ddPCR copy number assay results for all agricultural commodities with 1SST-6G-FFT as target, and *Lp*Cul4 as endogenous reference is presented in Figure 4. For the majority of commodities, copy number averages in qPCR and ddPCR were around one. However, the silage result for qPCR was three times higher than that of ddPCR (0.4 and 1.5 copies respectively). Reproducibility of the qPCR and ddPCR assays was evaluated using the coefficient of variance (CV) in lines (Figure 4). ddPCR revealed improved reproducibility (CVs between 0.9 and 17.6%) compare with those of qPCR (CVs between 5.1 and 41%).

### LoD and LoQ

**FIGURE 5:**
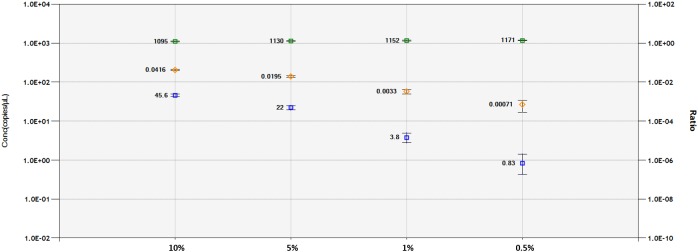
Limit of detection and limit of quantification of 1SST-6G-FFT construct (FAM in blue), and *Lp*Cul4 (HEX in green) as reference gene using droplet digital PCR. Blue and green plots indicate the concentration of positive droplets (counts/μL; *Y*-axis on the left side) for the 1SST-6G-FFT and *Lp*Cul4 sequences, respectively, and the average concentration is shown the left side of the plot. Orange plots show the copy number ratio of 1SST-6G-FFT (*Y*-axis on the right side), which was calculated through the average concentration of FAM-positive droplets divided by HEX-positive droplets. Error bars indicate the Poisson 95% confidence intervals for each measurement.

A DNA dilution test was performed with ddPCR and qPCR-based technologies for the 1SST-6G-FFT and *hph* sequences, using the *Lp*Cul4 probe as reference (Figure 5 and Supplementary Figures S6, S7). Genomic DNA from transgenic leaves (event 10) and non-transgenic leaves (FLP 418-20) were adjusted to 10, 5, 1, and 0.5 ng for the 20 μl PCR mixture. In qPCR, using primers and probes for 1SST-6G-FFT reliable detection was achieved at 10 and 5% dilution, while for *hph* detections up to 1% was obtained. Both exogenous constructs showed similar results in the ddPCR assay, the number of positive droplets for the target gene decreased relatively linearly, compared with the reference. When analyzing 1SST-6G-FFT with Cul4, concentration of the reference gene was 1095, 1130, 1152, and 1171 copies/μl, while for the target gene was 45.6, 22.0, 3.8 and 0.83 copies/μl for 10, 5, 1 and 0.5% respectively (Figure 5). Similarly, when analyzing *hph* with *Lp*Cul4, concentration of the reference gene was 1096, 1097, 1117, and 1132 copies/μl, while for the target gene was 47.3, 22.5, 3.9, and 1.5 copies/μl for 10, 5, 1 and 0.5% respectively (Supplementary Figure S7). The ratios (target/reference) for 1SST-6G-FFT were 0.0416, 0.0195, 0.0033, and 0.00071 and those for *hph* were 0.0432, 0.0205, 0.003, and 0.00013 with 10, 5, 1, and 0.5% of DNA respectively.

## Discussion

Development of detection techniques for new transgenic events are a prerequisite to comply with traceability and labeling requirement of GM plants for commercial realize (European Parliament, 2003) and in the management of co-existence frameworks in agricultural production systems (Putnam et al., 2016; Smith and Spangenberg, 2016). Therefore, development and validation of techniques capable of detecting, and quantifying the presence of GM forage crops at the farm gate, the processor, and the retailer level are necessary. In forage species intended for animal feeding, where human consumption is indirect, studies have focused on the digestive fate of recombinant DNA and proteins. Most of the studies concluded that transgenic DNA was broken down in the digestive system of animals (Flachowsky and Reuter, 2017). Therefore, efforts in GM detection for feedstuff species should not focus on milk or meat, but rather on other agricultural products (Smith and Spangenberg, 2016).

While all applicants for commercial release of transgenic products are required to provide a reliable method to detect the transgene below the tolerant threshold of each legislation, not all consider evaluating the method in different agricultural products. Determination of sample matrixes to be tested should be assessed in a case-by-case basis. For transgenic high-energy ryegrass, the most relevant tissues are fresh leaves, dry-leaves (hay), pollen, seeds, tillers and silage. The detection method of the transgene in herbage (fresh leaves and tillers) is essential, since it is the diet of gazing animals. Other factors such as traded material for sowing new pastures (seeds), gene flow (pollen) and the potential use of preservation and storage methods (hay and silage) are also needed to be considered. In the current study, a suitable DNA extraction method has been provided, using a commercially available kit.

Two main factors were evaluated during the sample preparation process, DNA quantity and quality. The first aspect refers to that sufficient DNA must be available to guarantee reliable detection of the transgene, and the second concerns the presence of undesired substances co-extracted with DNA, that may affect the accuracy of the detection method (Nadal et al., 2018). In the case study of high-energy ryegrass, DNA yield from pollen was relatively low, partially due to that pollen grains have a hard-outer shell, called exine (Lalhmangaihi et al., 2014). The presence of such tough coating in this matrix required additional DNA purification steps to obtain sufficient quality DNA. The DNA extraction kit used in this experiment was designed for plant tissue, and DNA extraction from fresh leaves, tillers, and even hay (dry leaves) was successfully performed. However, DNA from silage obtained after a 28-day fermentation process of the herbage showed the lowest level of quality, presumably due to endonucleotic enzyme activity during the fermentation (Tremblay et al., 2008).

Methods for detection of commercial transgenic forage crops are usually validated based on certified reference materials provided by the respective applicants. Transgenic and control reference material should be provided at the time of deposition of the dossier (Grelewska-Nowotko et al., 2018). For high-energy ryegrass, plasmid DNA with the exogenous cassette (1SST-6G-FFT and *hph*) was used as transgenic reference material. Due to the requirement for the endogenous reference gene to be quantitatively stable in all possible host genetic backgrounds (Marmiroli et al., 2008), the *Lp*Cul4 (Marín, 2009) single copy gene was selected.

Marín (2009) established the pattern of emergence and diversification of Cullin proteins in eukaryotes. It reveals that Cullin-RING ubiquitin ligases in animal, plant and fungi genomes, are ancient complex highly conserved and are likely to have a single copy status. In the present study, primers and probes for the Cul4 gene of perennial ryegrass, were designed, tested and the results corroborated that Cul4 is a suitable reference in ryegrass. In the same way a primer/probe set for the selectable marker (*hph*) were in house designed and tested. Although well-functioning primer/probe set for *hph* have been published previously (Collier et al., 2017), transgenic vectors may have small variations (Day et al., 2000).

Designing a set of primers and probe specific to the 1SST-6G-FFT sequence with acceptable amplification efficiency required an additional effort to be made, due to two main reasons. The first was that the coding sequence is constituted by two ryegrass endogenous genes (1SST and 6G-FFT) involved in the metabolic production of sucrose and fructan. Therefore, the only possible location for primers specific to the transgene was across junctions between two elements within the construct to avoid PCR amplification from the endogenous 1SST and 6G-FFT genes. The second, is that the nucleotide composition of insertion cassette was highly skewed toward guanine and cytosine.

Despite these limitations, the standard curve assays for the exogenous constructs and endogenous reference gene were reasonable. The low efficiency obtained with 1SST-6G-FFT in the probe-based assay (81.5%), is possibly related with formation of a complex secondary structure at GC-rich regions of the target amplicon, since GC content influences both optimal annealing temperatures and primer specificity (Mamedov et al., 2008). The optimal annealing temperature for the primer set, analyzed with a thermal gradient in ddPCR (Supplementary Figure S1), showed that to ensure GC-rich primers anneal stably to the template, higher annealing temperatures were necessary.

GM detection methods has depended on qPCR technologies, using either SYBR Green I or probe based fluorescence (Gao et al., 2016). The SYBR Green I assay showed ability to detect both 1SST-6G-FFT and *hph* in a range of agricultural products (Figure 2A and Supplementary Figure S4A). Even though this fluorescent detection method is less specific because it binds any double-stranded DNA, including undesired non-specific amplicons and primer dimers. SYBR Green I methods are gaining popularity as it enables adding an existing method to the already available screening, which can be run in a single 96-well plate (Broeders et al., 2015). Additionally, this technology is more cost-effective as no dye-labeled oligonucleotide probes are required.

Fluorescent probe-based qPCR is the preferred method when a quantitative analysis is required, since it reflects high accuracy, specificity and sensitivity (Gao et al., 2016). The evaluation of different agricultural products in this study, allowed to distinguish between different amplification products in the same reaction. Therefore, with this approach the differences in PCR efficiency and cycle threshold between reference gene and exogenous fragment amplicons were well defined. Although, the CV values in the qPCR results (Supplementary Table S1) were higher, compared to those obtained using ddPCR (Supplementary Table S2), in the first assay approach, the CVs were calculated from the Ct values, while in the second assay approach those were calculated based on the concentration of the positive droplets (copies/μL), so that the variability in the results was typically higher in qPCR than in ddPCR (ure 4). Compared with ddPCR, fluorescent probe-based qPCR requires reference material to calibrate the results, which adds more variation due to factors such as inhibitors and inherent measurement uncertainty (Köppel et al., 2015).

ddPCR can overcome those specificity issues in qPCR, through compartmentalization of a regular PCR mixture into millions of fractions (Lievens et al., 2016). A high sensitivity was observable in the evaluation of transgenic and non-transgenic agricultural products, and although the difference in DNA quality obtained from different commodities was still evident, results were clear and reliable (Figure 3). The high GC content of 1SST-6G-FFT, did not seem to have affected the amplification with this technique. However, the DNA quality of silage which is a product of partial degradation, was shown to affect the results. Similarly, Fearing et al. (1997) did not detect the CryIA protein from Bt maize silage, and they indicated that the ensiling process breaks down protein and fiber, rendering nutrients readily digestible to the ruminant animal. Hupfer et al. (1999) also demonstrated the effect of maize DNA degradation during the ensilage on the detectability of target sequences using qPCR, mainly due to the release of endogenous nucleases of the plant and/or exogenous nucleases of the microflora.

Transgenic DNA copy number assays using ddPCR have been reported in the major GM crops commercially available, such as maize (Dalmira et al., 2015; Xu et al., 2016; Collier et al., 2017; Grelewska-Nowotko et al., 2018), canola (Demeke and Eng, 2018), and soybean (Köppel et al., 2015; Iwobi et al., 2016; Wan et al., 2016). In the present study, all plants were previously selected to have a single copy of the transgene (ure 4). However, the number of transgenic copies in other studies varies depending on the type of transformation used. For instance, a study on maize to determinate copy number of *T-nos*, using *hmg* as the reference gene, found that the number of copies in different varieties were between 88.22 and 0.88, with a coefficient of variance from 14.8 to 2.3% (Dalmira et al., 2015). These results agree with the ddPCR results obtained in this study, which despite having predetermined single copy samples, CV varies between 0.9 and 17.6%.

The minimum labeling threshold on GMO content in feed and foodstuffs are 0.9% in European Union (EU), 1% in New Zealand and Australia, 3% in Korea, and 5% in the United States (US) and Japan (Gao et al., 2016). Although, qPCR showed to be effective, ddPCR achieved a reliable detection of both exogenous constructs below the threshold of all jurisdictions. Similarly, ddPCR studies have showed reliable transgene detection of maize, soybean and canola at 1% (Dalmira et al., 2015; Demeke et al., 2016; Wan et al., 2016 respectively). qPCR can be a convenient method for qualitative detection with lower cost, in terms of instruments and reagents compared with ddPCR, but an accurate detection in qPCR can be limited when the target is present at low concentrations.

## Conclusion

Detection and quantification of all transgenic pasture-based feed products should be assessed in all relevant agricultural commodities, since there is a high variability in DNA quantity and quality extracted from them, which affects its subsequent quantification. Determination of agricultural commodities to test, should consider factors such as storage, and/or processing. qPCR may be more suited for routine screening as it is very cost-efficient, while ddPCR may be more suitable for quantitative analysis as it allows an absolute quantification of the target sequence.

For high-energy ryegrass, detection of associated selectable marker genes such as *hph* would be advisable if using qPCR to avoid the GC-rich nature of the specific transgene, and both targets (1SST-6G-FFT and *hph*) can be used in ddPCR. To comply with labeling requirements in Europe as well as set global standards, ddPCR should be used to guarantee a reliable detection below the minimal threshold. However, in all other legislations with feed traded at a national level, qPCR with SYBR Green I can be used for general screening of a small number of targets common to numerous events (such as *hph*), Fluorescent probe-base qPCR to quantify event copy number and ddPCR can be used to support the results if need. For feed products intended to be traded globally, a standard method proven to be sensitive and reliable such us ddPCR should be considered.

This document provides guidance to Plant biotechnologists working on pasture based crops to assess GM crops in different agricultural commodities and with complex transgene sequences, such as cisgenic sequences (endogenous gene) that are also GC rich. However, studies must be carried out following a case-by-case approach for the evaluation of GM feed.

## Author Contributions

PG and HS conducted the experimental work and data analysis. NC, KS, and GS provided overall project leadership. PG, NC, and HS prepared the primary drafts of the manuscript and contributed to finalization of the text. PG, HS, KS, and GS co-developed interim and final drafts of the manuscript. All authors have read and approved the final manuscript.

## Conflict of Interest Statement

The authors declare that the research was conducted in the absence of any commercial or financial relationships that could be construed as a potential conflict of interest.

## Footnotes

1 http://bioinfo.ut.ee/primer3-0.4.0/
