# Supplementary material for: Development and Application of Droplet Digital PCR Tools for the Detection of Transgenes in Pastures and Pasture-Based Products
Source: Front Plant Sci. 2019 Jan 8;9:1923. doi: 10.3389/fpls.2018.01923 (PMC6331530; doi:10.3389/fpls.2018.01923)
Supplement: Supplementary file 1 [file Data_Sheet_1.pdf]

Supplement legends

Table S1. Cycle threshold mean (Ct), standard deviation (SD), and coefficient of variation (CV%) using qPCR with probe-based fluorescent. GOI refers to gene of interest, and plus (+) and minus (-) signs indicate positive (+) and negative (-) for GOI.

Table S2. Concentration (Conc, copies/uL), standard deviation (SD), and CV using ddPCR with fluorescent probe-based. GOI refers to gene of interest, and plus (+) and minus (-) signs indicate positive (+) and negative (-) for GOI.

Table S3. Oligonucleotide primer and probe sequences of the exogenous constructs and endogenous reference gene.

Fig. S1. Standard Curve assay for *Lp*Cul4 (**a**), 1SST-6G-FFT (**b**), and *hph* (**c**), using qPCR with SYBR Green I fluorescence.

Fig. S2. Standard curve assay for 1SST-6G-FFT construct (FAM in blue) and *Lp*Cul4 (HEX in green) (**a**), and *hph* (FAM in blue) and *Lp*Cul4 (HEX in green) (**b**) using qPCR with fluorescent probes.

Fig. S3. 1D fluorescence amplitude plot of the ddPCR assay using a gradient of temperature (65-55°C) for 1SST-6G-FFT construct (FAM in blue) and *Lp*Cul4 (HEX in green) (**a**), and *hph* (FAM in blue) and *Lp*Cul4 (HEX in green) (**b**).

Fig. S4

Detection and quantification of *hph* construct, using qPCR with SYBR Green I fluorescence (**a**), and fluorescent probes (**b**). In the probe-base assay, the target construct (*hph,* FAM) is shown in blue and the reference gene (*Lp*Cul4, HEX) in green. The Y-axis shows relative fluorescence unit (RFU), and the X-axis denotes PCR cycle number.

Fig. S5

Detection and quantification of *hph* construct (FAM in blue) using ddPCR. (**a**) 1D fluorescence amplitude plot, where set threshold is shown with a pink line, blue plots indicate presence of the *hph* sequence in the droplet, and grey plots indicate absence of the sequence. (**b**) Ratio of *hph* construct and *Lp*Cul4 (HEX). UT and T stand for untransformed and transformed, respectively. Error bars indicate the Poisson 95% confidence intervals for each measurement.

Fig. S6

Limit of detection and limit of quantification of 1SST-6G-FFT construct (FAM in blue) (**a**) and *hph* (FAM in blue) (**b**) using *Lp*Cul4 (HEX in green) as the reference gene in a qPCR probe-based assay.

Fig. S7

Limit of detection and limit of quantification of *hph* construct (FAM in blue) and *Lp*Cul4 (HEX in green) as reference gene, using droplet digital PCR. Blue and green plots indicate the concentration of positive droplets (counts/µL; Y-axis on the left side) for the *hph* and *Lp*Cul4 sequences, respectively, and the average concentration is shown the left side of each plot. Orange plots show the copy number ratio of *hph* (Y-axis on the right side), which was calculated through the average concentration of FAM-positive droplets divided with that of HEX-positive droplets. Error bars indicate the Poisson 95% confidence intervals for each measurement.

Fig. S8

Melting curve assay and TapeStation of qPCR products for 1SST-6G-FFT (a and b respectively), and *hph* (c and d) and using SYBR Green I fluorescence.

Table S1

Tissue	GOI	Primers	Mean Ct	SD	CV (%)
Leave	-	1SST-6G-FFT	35.0	0.0	0.0
		LpCull4	24.0	0.4	1.7
Leave	+	1SST-6G-FFT	25.7	0.7	2.6
		LpCull4	24.2	0.6	2.5
Tiller	-	1SST-6G-FFT	34.9	0.3	0.8
		LpCull4	24.5	0.6	2.6
Tiller	+	1SST-6G-FFT	25.6	0.8	3.2
		LpCull4	24.2	0.7	2.9
Silage	-	1SST-6G-FFT	34.4	0.8	2.3
		LpCull4	27.7	1.6	5.7
Silage	+	1SST-6G-FFT	30.7	1.0	3.3
		LpCull4	28.2	0.8	2.8
Hay	-	1SST-6G-FFT	35.0	0.0	0.0
		LpCull4	23.1	0.6	2.8
Hay	+	1SST-6G-FFT	24.2	0.6	2.7
		LpCull4	23.1	0.6	2.6
Seed	-	1SST-6G-FFT	35.0	0.0	0.0
		LpCull4	24.9	0.2	1.0
Seed	+	1SST-6G-FFT	29.0	1.3	4.5
		LpCull4	25.0	0.4	1.6
Pollen	-	1SST-6G-FFT	35.0	0.0	0.0
		LpCull4	23.4	1.2	5.1
Pollen	+	1SST-6G-FFT	27.0	0.5	1.7
		LpCull4	24.6	1.6	6.5

Table S2

Tissue	GOI	Primers	Conc(copies/uL)	SD	CV (%)
Leave	-	1SST-6GFFT	3.8	0.1	1.8
		LpCull4	474.6	14.7	3.1
Leave	+	1SST-6GFFT	287.8	5.8	2.0
		LpCull4	463.4	22.9	4.9
Tiller	-	1SST-6GFFT	0.3	0.0	5.8
		LpCull4	495.8	8.9	1.8
Tiller	+	1SST-6GFFT	305.3	2.2	0.7
		LpCull4	547.6	8.0	1.5
Silage	-	1SST-6GFFT	3.8	0.2	4.0
		LpCull4	25.9	0.5	2.1
Silage	+	1SST-6GFFT	26.4	0.5	1.9
		LpCull4	22.0	1.0	4.5
Hay	-	1SST-6GFFT	0.4	0.0	8.5
		LpCull4	774.2	3.7	0.5
Hay	+	1SST-6GFFT	381.6	5.7	1.5
		LpCull4	754.2	25.8	3.4
Seed	-	1SST-6GFFT	2.6	0.2	7.1
		LpCull4	303.6	5.8	1.9
Seed	+	1SST-6GFFT	108.0	1.7	1.6
		LpCull4	316.3	5.8	1.8
Pollen	-	1SST-6GFFT	71.3	1.0	1.4
		LpCull4	162.5	4.0	2.5
Pollen	+	1SST-6GFFT	42.0	2.3	5.4
		LpCull4	57.6	3.0	5.2

Table S3

Target	Primer	Sequence (5′–3′)	Product (bp)
Endogene	*Lp*Cul4-f	CCGATTAAGCCAGCGGATA	122
	*Lp*Cul4-r	CATCTCTTTCATGGCTGTCA	
	*Lp*Cul4-P (HEX)	ACCTGGAGAGAGACCGAAGTA	
Exogene	*Lp*1SST-6G-FFT -f	AGGCATAGCCCAGCTAGTTA	137
	*Lp*1SST-6G-FFT -r	CGCGTACGCATAAGGAAGCA	
	*Lp*1SST-6G-FFT -P(FAM)	CCCGCGGTGAATTCATGGAGTCCC	
Exogene	*Hph*-f	ATTTCGGCTCCAACAATGTC	105
	Hph-r	AGATGTTGGCGACCTCGTAT	
	Hph-P (FAM)	TTGACTGGAGCGAGGCGATGTTC	

Figure S1

a

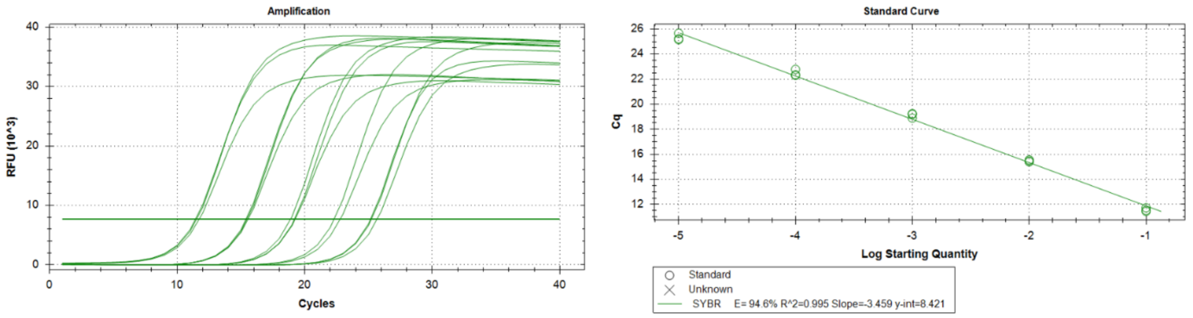

b

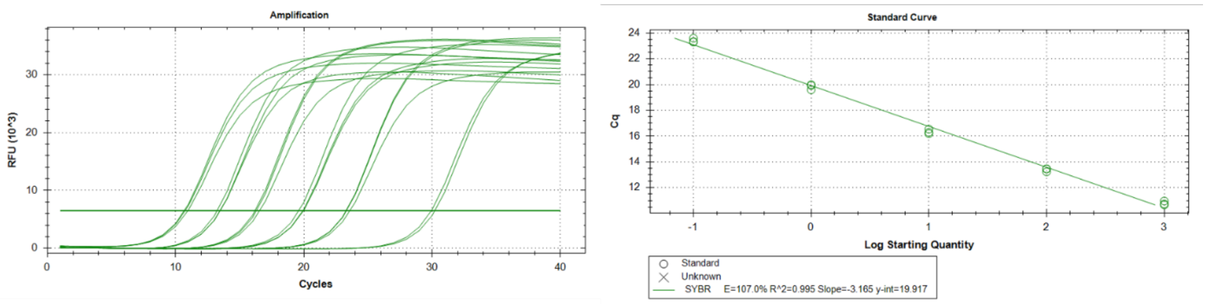

c

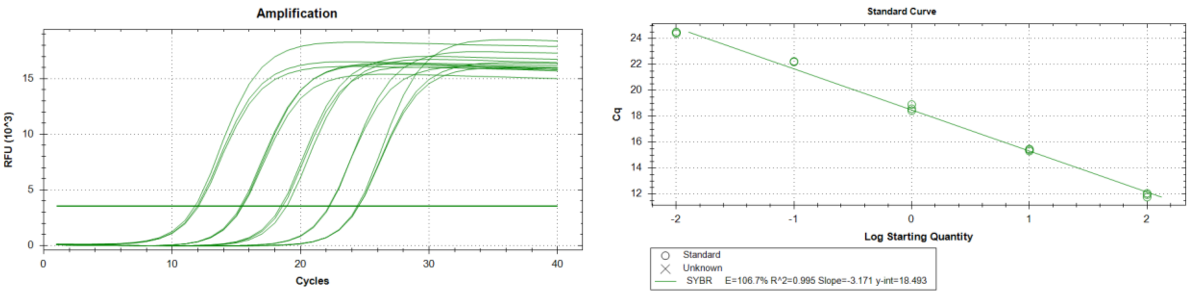

Figure S2

a

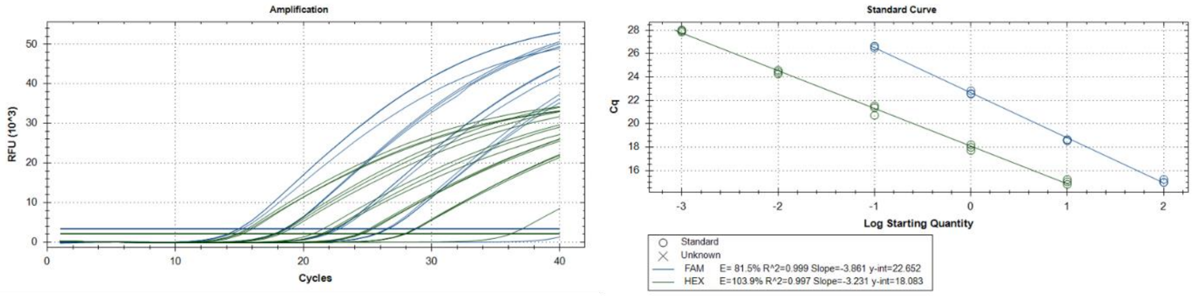
Amplification
RFU (10^3)
Cycles
Standard Curve
Cq
Log Starting Quantity
Standard
Unknown
FAM E= 81.5% R^2=0.999 Slope=-3.861 y-int=22.652
HEX E=103.9% R^2=0.997 Slope=-3.231 y-int=18.083

b

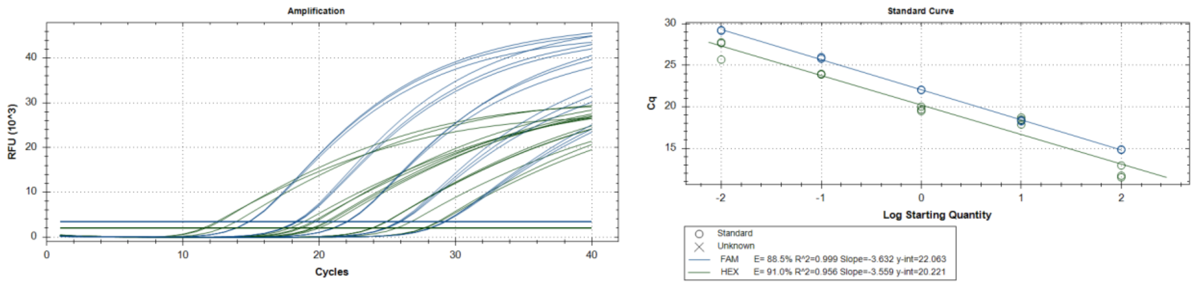
Amplification
RFU (10^3)
Cycles
Standard Curve
Cq
Log Starting Quantity
Standard
Unknown
FAM E= 88.5% R^2=0.999 Slope=-3.632 y-int=22.063
HEX E= 91.0% R^2=0.956 Slope=-3.559 y-int=20.221

Figure S3

a

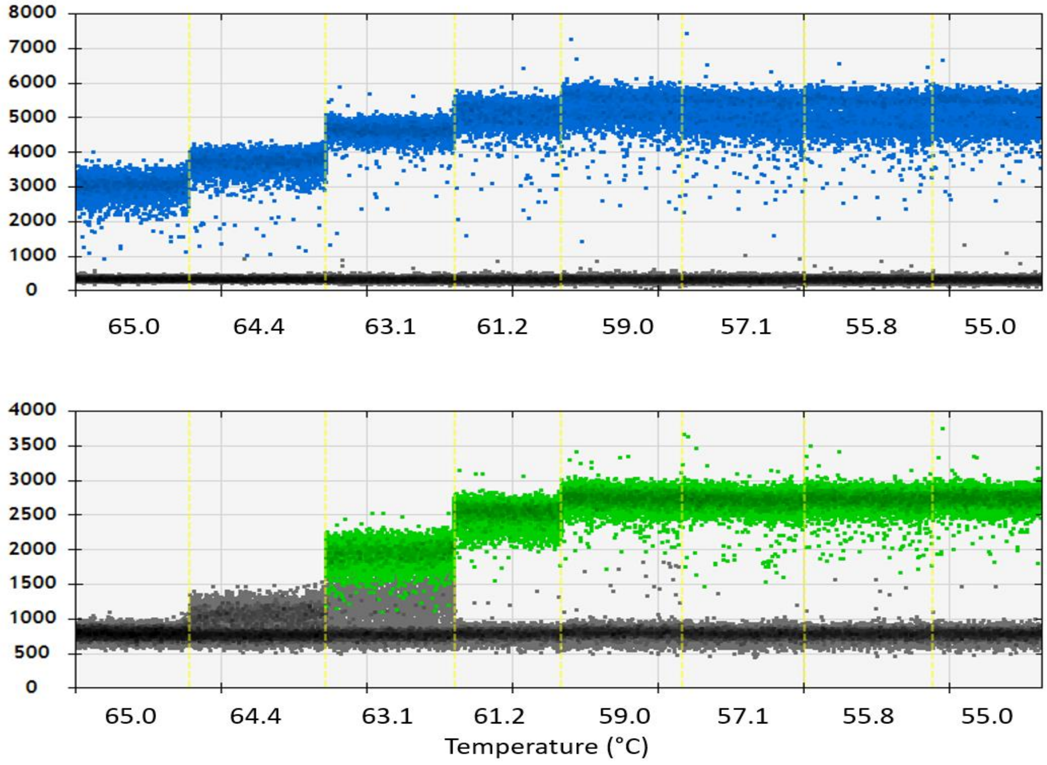
8000
7000
6000
5000
4000
3000
2000
1000
0
65.0
64.4
63.1
61.2
59.0
57.1
55.8
55.0
4000
3500
3000
2500
2000
1500
1000
500
0
65.0
64.4
63.1
61.2
59.0
57.1
55.8
55.0
Temperature (°C)

b

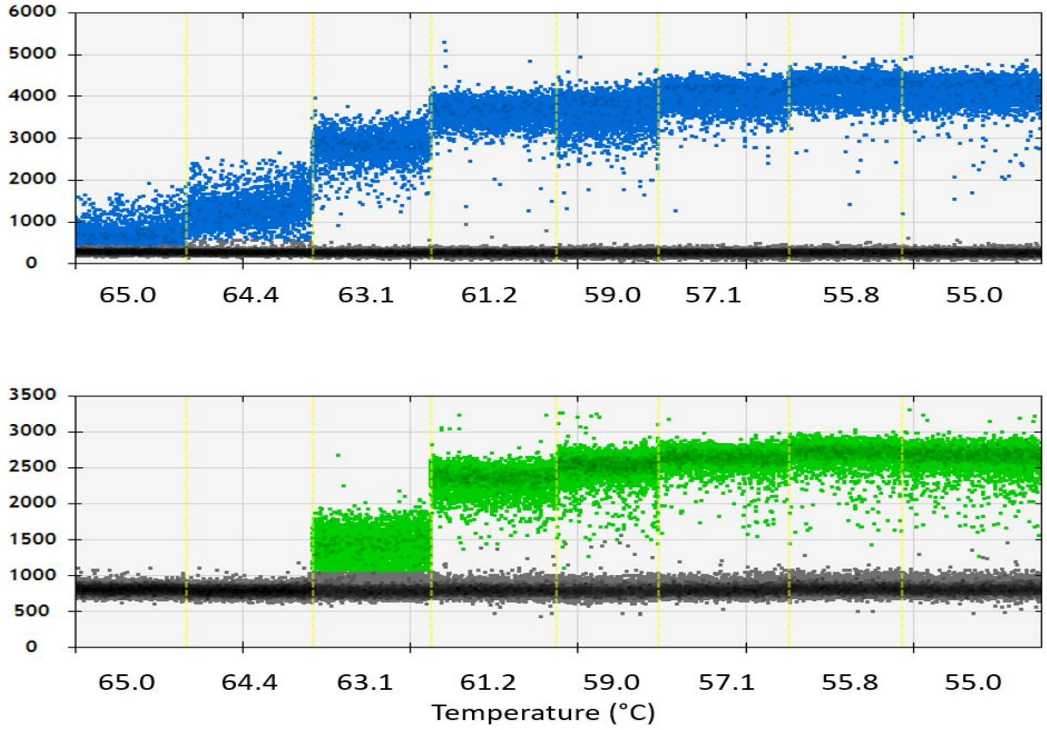
6000
5000
4000
3000
2000
1000
0
65.0
64.4
63.1
61.2
59.0
57.1
55.8
55.0
3500
3000
2500
2000
1500
1000
500
0
65.0
64.4
63.1
61.2
59.0
57.1
55.8
55.0
Temperature (°C)

Figure S4

a

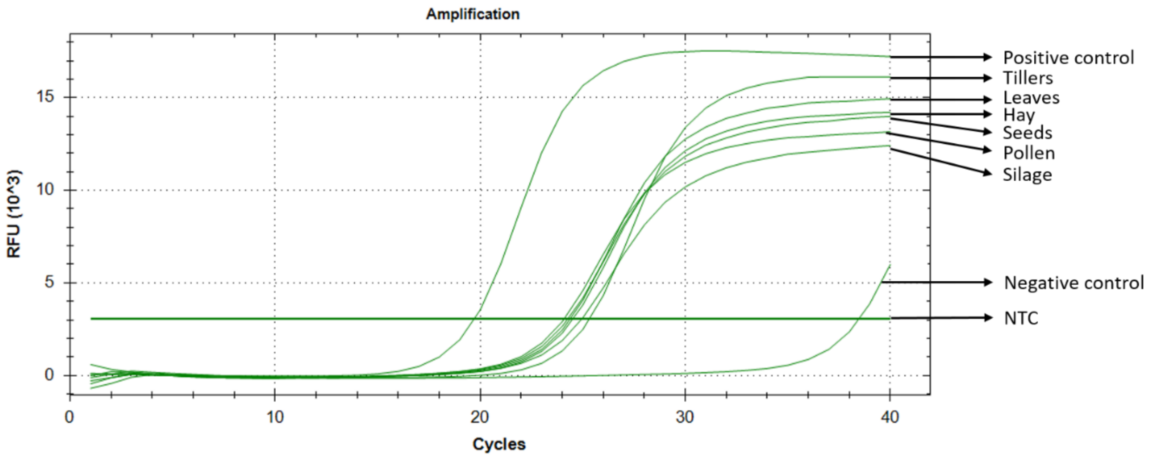
Amplification
RFU (10^3)
0
5
10
15
Cycles
0
10
20
30
40
Positive control
Tillers
Leaves
Hay
Seeds
Pollen
Silage
Negative control
NTC

b

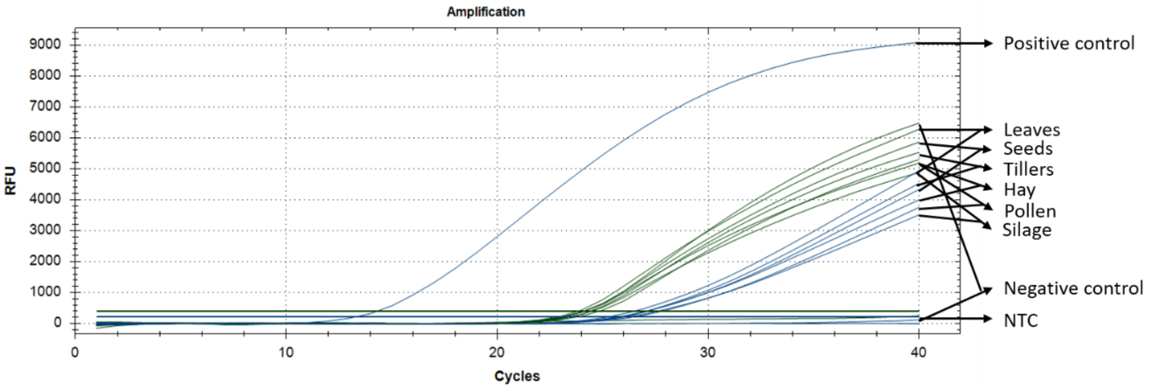
Amplification
RFU
0
1000
2000
3000
4000
5000
6000
7000
8000
9000
Cycles
0
10
20
30
40
Positive control
Leaves
Seeds
Tillers
Hay
Pollen
Silage
Negative control
NTC

Figure S5

a

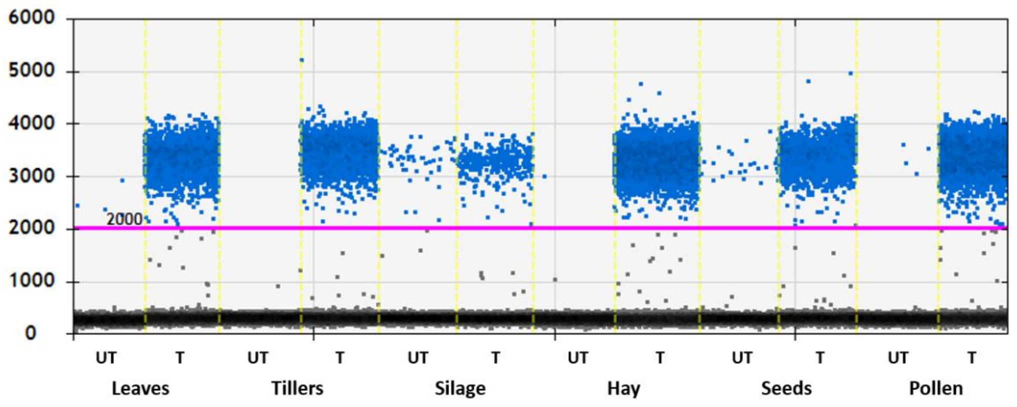
6000
5000
4000
3000
2000
1000
0
2000
UT T UT T UT T UT T UT T UT T
Leaves Tillers Silage Hay Seeds Pollen

b

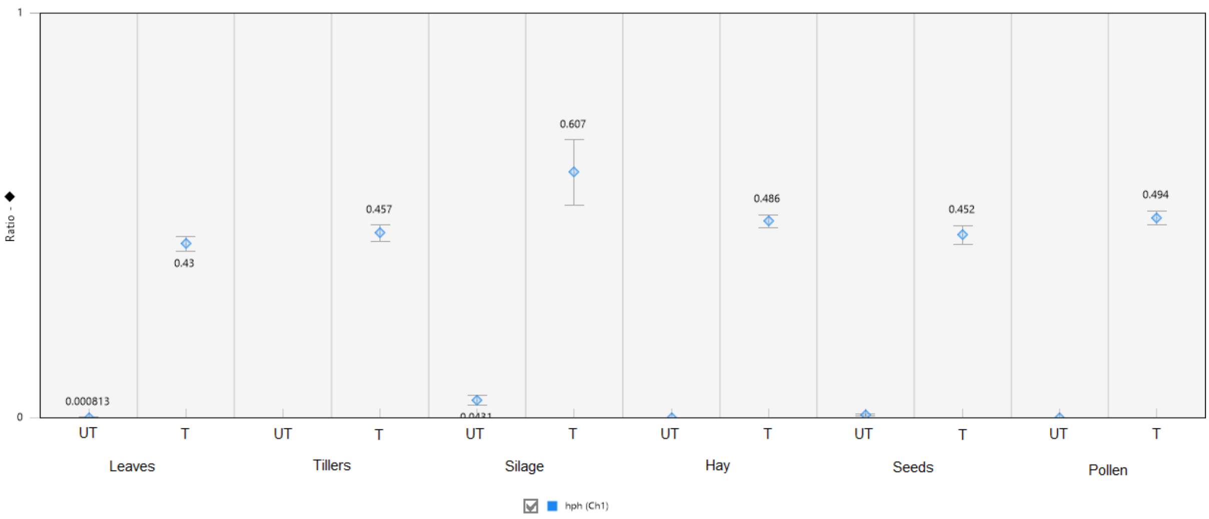
1
0
Ratio -
0.000813
0.43
0.457
0.607
0.486
0.452
0.494
UT T UT T UT T UT T UT T UT T
Leaves Tillers Silage Hay Seeds Pollen
hph (Ch1)

Figure S6

a

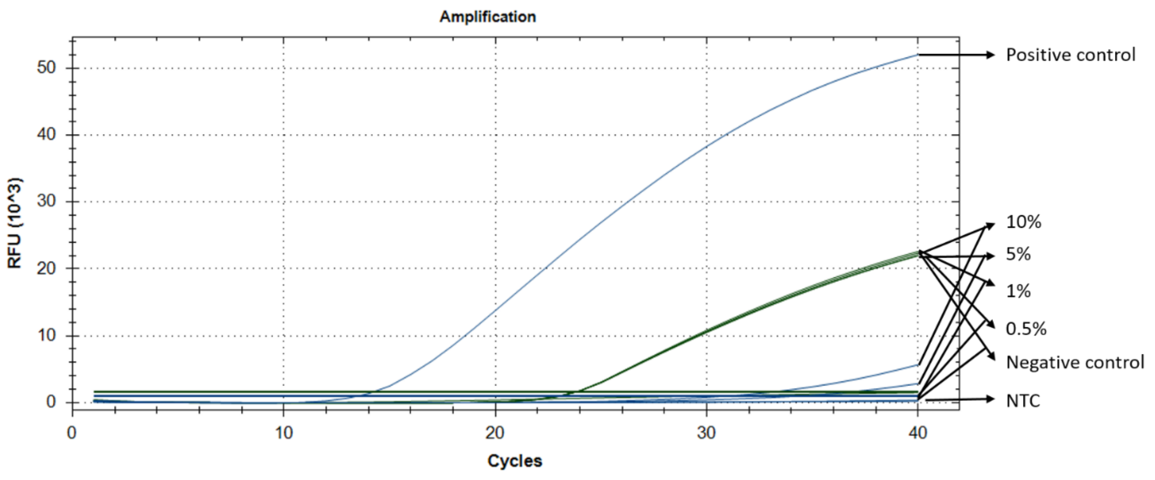

Amplification
RFU (10^3)
50
40
30
20
10
0
0
10
20
30
40
Cycles
Positive control
10%
5%
1%
0.5%
Negative control
NTC

b

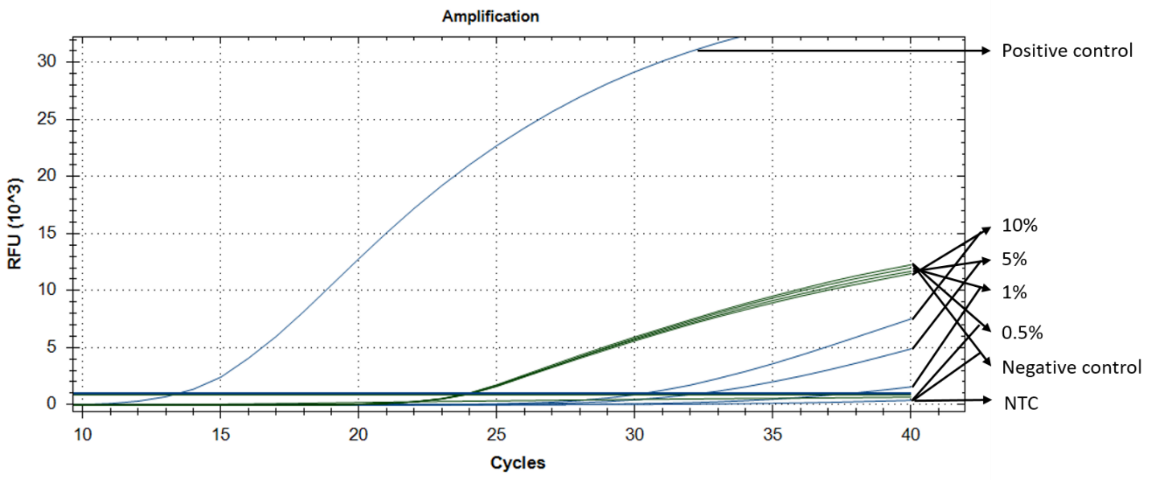

Amplification
RFU (10^3)
30
25
20
15
10
5
0
10
15
20
25
30
35
40
Cycles
Positive control
10%
5%
1%
0.5%
Negative control
NTC

Figure S7

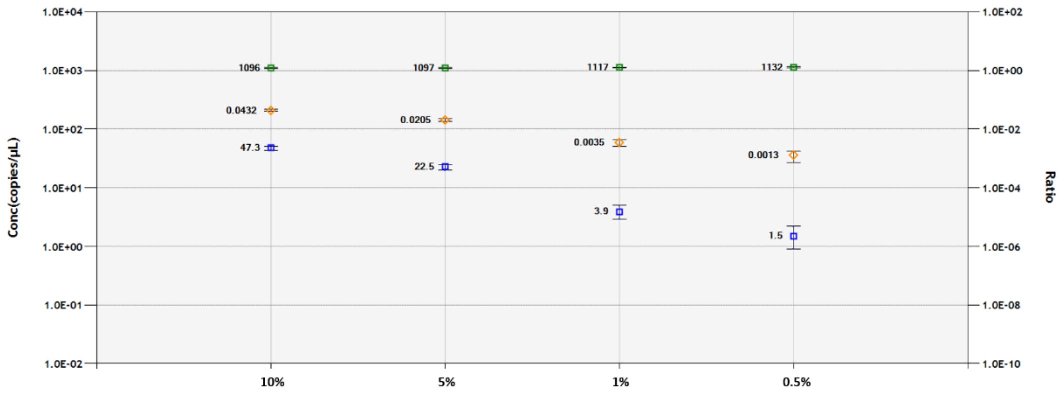

1.0E+04
1.0E+03
1.0E+02
1.0E+01
1.0E+00
1.0E-01
1.0E-02
Conc(copies/µL)
1.0E+02
1.0E+00
1.0E-02
1.0E-04
1.0E-06
1.0E-08
1.0E-10
Ratio
1096
1097
1117
1132
0.0432
0.0205
0.0035
0.0013
47.3
22.5
3.9
1.5
10%
5%
1%
0.5%

Figure 8

a

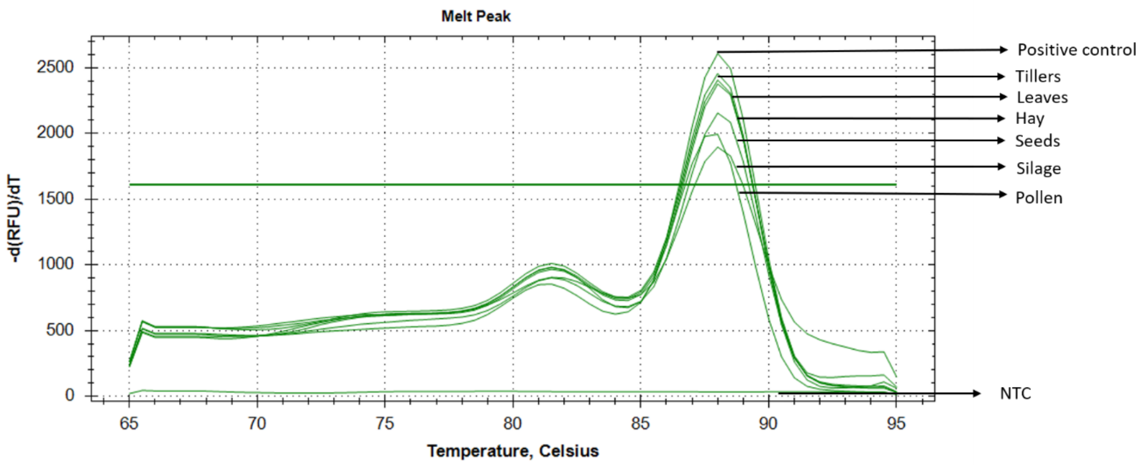
Melt Peak
-d(RFU)/dT
2500
2000
1500
1000
500
0
65
70
75
80
85
90
95
Temperature, Celsius
Positive control
Tillers
Leaves
Hay
Seeds
Silage
Pollen
NTC

b

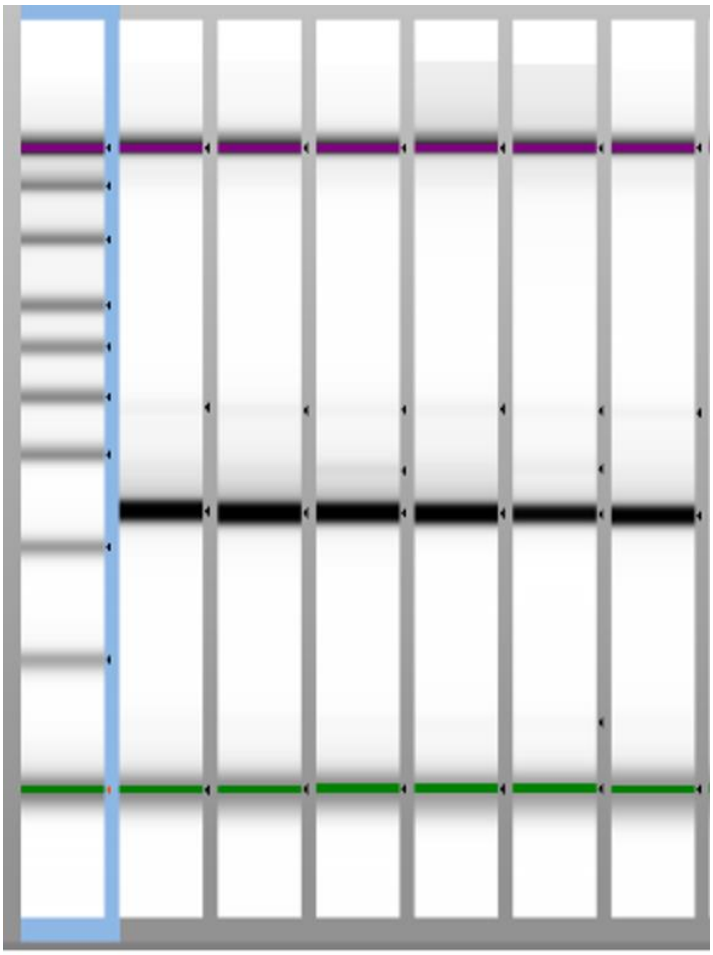
Ladder Leaves Tillers Hay Seeds Silage Pollen

c

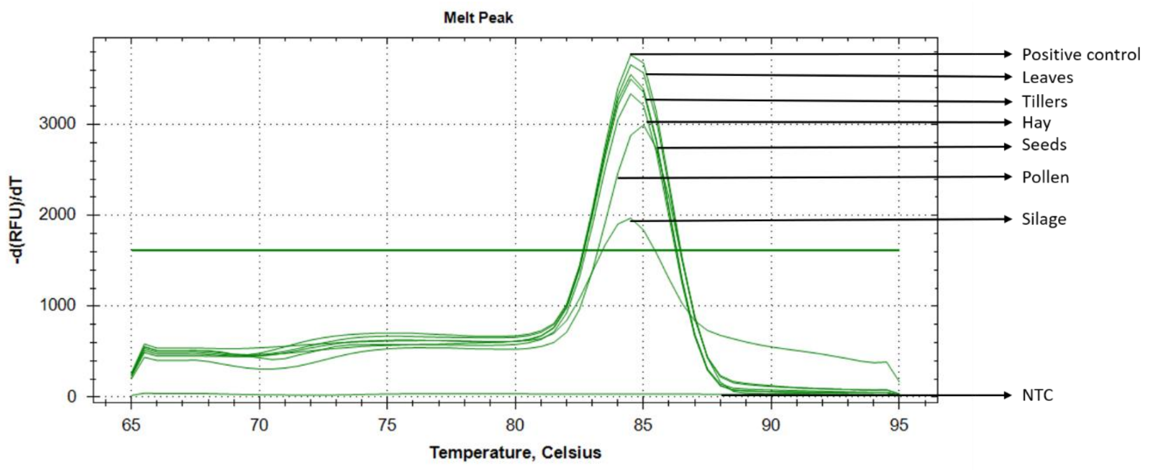
Melt Peak
-d(RFU)/dT
3000
2000
1000
0
65
70
75
80
85
90
95
Temperature, Celsius
Positive control
Leaves
Tillers
Hay
Seeds
Pollen
Silage
NTC

d

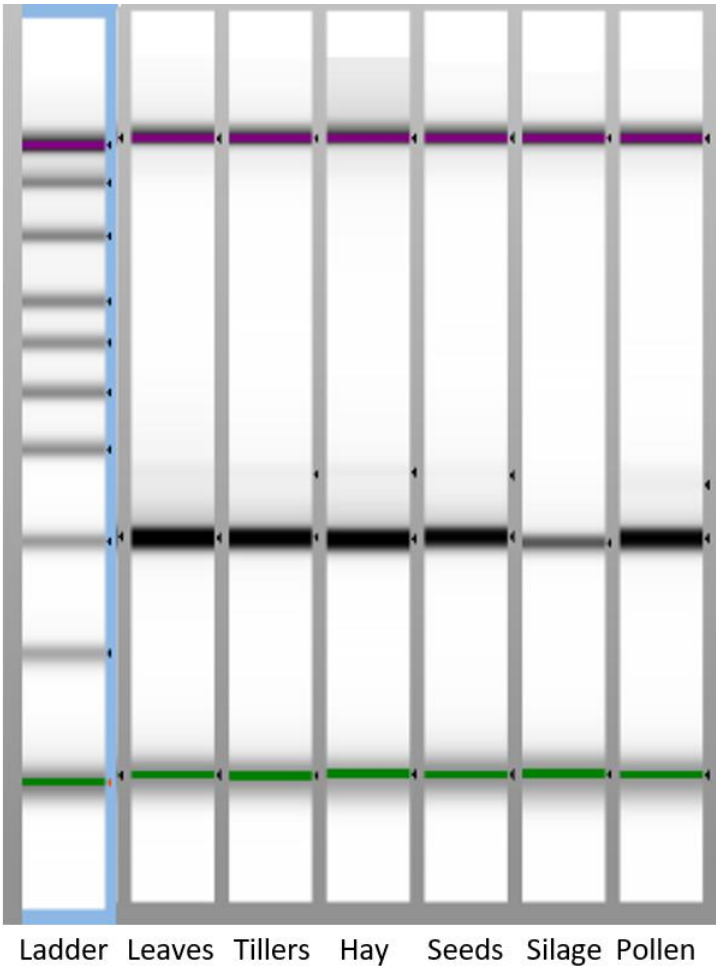
Ladder
Leaves
Tillers
Hay
Seeds
Silage
Pollen
